# Sr isotope composition of Golden Delicious apples in Northern Italy reflects the soil ^87^Sr/^86^Sr ratio of the cultivation area

**DOI:** 10.1002/jsfa.10399

**Published:** 2020-04-25

**Authors:** Agnese Aguzzoni, Michele Bassi, Emanuela Pignotti, Peter Robatscher, Francesca Scandellari, Werner Tirler, Massimo Tagliavini

**Affiliations:** ^1^ Free University of Bozen‐Bolzano Bozen‐Bolzano Italy; ^2^ Laimburg Research Centre Auer‐Ora Italy; ^3^ Eco‐Research srl Bozen‐Bolzano Italy

**Keywords:** *Malus* × *domestica* Borkh., isotope ratio, soil‐derived marker, geographical origin, geological features

## Abstract

**BACKGROUND:**

Apples have a leading role in the Italian fruit sector, and high‐quality apples, including the Golden Delicious variety, are cultivated mainly in the Northern mountain districts. In the present study, Golden Delicious apples from PDO (Protected Designation of Origin) and PGI (Protected Geographical Indication) cultivation districts were characterized according to their Sr isotope composition and compared with apples from other Northern Italian districts.

**RESULTS:**

Apples collected in two consecutive years (2017 and 2018) confirmed the low annual variability of the ^87^Sr/^86^Sr ratio. The isotope ratio of apples was highly correlated with that of the soil extracts of the respective orchards. Statistical differences were highlighted between cultivation districts. However, because similar geological features characterized some areas, their ratios overlapped and a complete separation of the districts was not possible.

**CONCLUSION:**

The ^87^Sr/^86^Sr ratio is an excellent marker for studies of food traceability because it retains the information about the place of origin. However, its strength is limited when comparing products from cultivation areas sharing similar geological features. In the perspective of geographical traceability, a multichemical characterization can overcome the limits of single‐parameter approach. © 2020 The Authors. *Journal of The Science of Food and Agriculture* published by John Wiley & Sons Ltd on behalf of Society of Chemical Industry.

## INTRODUCTION

In the European Union (EU), apples (*Malus* × *domestica* Borkh.) are the most important fruits in terms of harvested product (average annual production of approximately 12 million of Mg in the triennium 2015–2017).^1^ With a total amount of approximately 2.3 million of Mg year^−1^ (2015–2017), Italy ranks seventh as an apple producer at the global scale, but ranks third with respect to export, which, in 2017 accounted for more than US $950 million.^2^, ^3^ Golden Delicious is the main variety both on a continental and national scale, although its acreage has decreased in the recent years. In Italy, its cultivation is widespread in the Northern regions, especially in the alpine districts of South Tyrol and Val di Non (Trentino‐South Tyrol region) and Valtellina (Lombardia region).

About two‐thirds of Italian apples come from Trentino‐South Tyrol. As a result of microclimate and geological conditions, the region represents an excellent cultivation area for apples. In the triennium 2015–2017, apple production reached impressive goals, with an average of approximately 1 000 000 and 420 000 Mg year^−1^ of harvested apples in South Tyrol and Trentino, respectively. During the 20th Century, apple cultivation expanded largely also in Valtellina (Lombardia) and, in the last few decades, it reached 35 000 Mg year^−1^.

Between 2003 and 2010, several apple varieties, including Golden Delicious, cultivated in these three areas of Northern Italy were granted with the EU Geographical Indications (GIs). In 2003, apples from Val di Non received the PDO label (‘Mela Val di Non’, EC Regulation 1665/2003), whereas those from South Tyrol and Valtellina obtained the PGI label, respectively, in 2005 (‘Mela Alto Adige‐Südtiroler Apfel’, EC Regulation 1855/2005) and 2010 (‘Mela di Valtellina’, EC Regulation 171/2010). These labels certify the link between unique features of the product and the place of origin. Moreover, they ensure the consumers the compliance with specific production guidelines, which also define a well‐established registration system along the whole production chain.

Analytical methods are important tools for protecting the authenticity and the reputation of high‐quality horticultural products labeled with GIs, as in the case of apples. Among the available methods, the analysis of the ^87^Sr/^86^Sr ratio has been largely applied to link a product with its geographical origin because it provides an almost unique fingerprint linked to the soil where trees grow.^4^, ^5^ The ^87^Sr/^86^Sr ratio of a crop is directly related to that of the Sr‐bioavailable fraction in the soil, which mainly depends and varies according to the geolithological features of the growing area.^6^, ^7^, ^8^ Its high potential as soil‐derived traceability marker to distinguish different types of horticultural products according to their origin, either alone or coupled with other analytical methods has been demonstrated in several studies.^9^, ^10^, ^11^, ^12^, ^13^

The present study aimed at applying ^87^Sr/^86^Sr analysis to characterize the Italian PDO and PGI Golden Delicious apples in comparison with fruits of the same variety from other areas of Northern Italy where no GIs for apples are registered (non‐GI apples from Emilia Romagna, Lombardia, Piemonte and Veneto). The annual variability of the ^87^Sr/^86^Sr ratio in apple samples was verified comparing the ^87^Sr/^86^Sr ratio of apples collected from the same orchards in two consecutive years (2017 and 2018). Moreover, the apple ^87^Sr/^86^Sr ratio was evaluated in relation to the ^87^Sr/^86^Sr ratio of the bioavailable Sr fraction extracted from the soil sampled in each orchard and the data were interpreted according to the local geolithological information.

## MATERIALS AND METHODS

### Reagents

Nitric acid (Merck, Darmstadt, Germany) and high purity deionized water (18.2 MΩ cm) (Elix; Millipore, Billerica, MA, USA), additionally purified through a sub‐boiling duoPur distillation system (Milestone, Sorisole, Italy), were used throughout the entire experiment and analytical work. Ammonium nitrate and Suprapur® hydrogen peroxide were purchased from Sigma‐Aldrich (St Louis, MO, USA). Mono‐elemental certified standards of rubidium (Rb) and strontium (Sr) were purchased from ULTRA Scientific (North Kingstown, RI, USA); calcium (Ca) from Agilent Technologies (Agilent Technologies Inc., Santa Clara, CA, USA); and germanium (Ge), scandium (Sc) and yttrium (Y) from Merck. Quality controls (QCs) were prepared diluting the TMDA‐64.3 certified reference material (LabService Analytica Srl, Anzola dell'Emilia, Italy) for the inductively coupled plasma mass spectrometer (ICP‐MS) and the SRM 987 (NIST, Gaithersburg, MD, USA) with a certified Sr isotope composition for the multicollector ICP‐MS (MC ICP‐MS). Strontium separation was accomplished using a strontium–selective resin (SR‐B100‐S, particle size 50–100 μm) purchased from TrisKem International (Bruz, France). Cellulose acetate filters (0.45 μm) and polypropylene disposable tubes were purchased from Vetrotecnica (Padova, Italy). Hydrophilic polytetrafluoroethylene (PTFE) filters (0.45 μm) were obtained from Thermo Fisher Scientific (Waltham, MA, USA). All the disposable vessels and tubes were soaked for 24 h with a nitric acid solution (0.7 mol L^–1^) and then rinsed with high‐purity water before use. Reagents and chemicals were stored in accordance with the respective manufacturer’s instructions.

### Sampling sites

**Figure 1:**
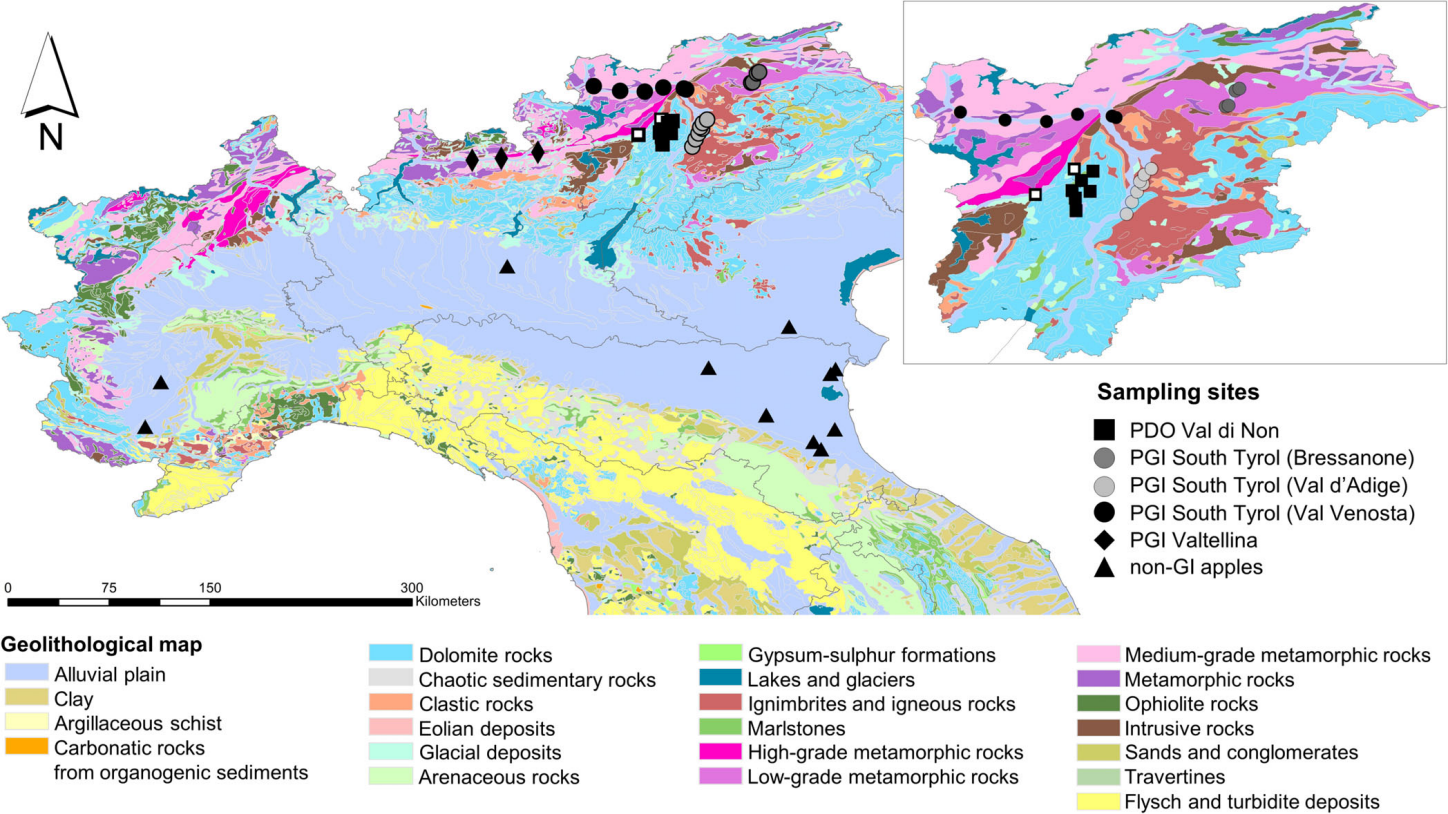
Distribution of the orchards (2017 sampling campaign) on a geological map (source: http://www.pcn.minambiente.it). Data were spatially referenced to the image using ArcGIS (https://www.arcgis.com). Data are grouped according to the cultivation district. The enlarged image illustrates the sampling sites in Trentino – South Tyrol. Open squares represent the two orchards in Val di Non excluded from the analysis of variance among cultivation districts.

Two sampling campaigns were performed to collect apple and soil samples between August and September in both 2017 and 2018 (Fig. 1 and Table 1). All selected orchards were cultivated with the same apple variety (cv. Golden Delicious, rootstock M9) and the trees were in full production. Fruit sampling was performed in the 2‐week period before harvest (August to September). The apple orchards included in the study were located in five regions of Northern Italy: Emilia Romagna, Lombardia, Piemonte, Trentino‐South Tyrol and Veneto. The number of orchards per region varied according to the extension of the cultivation area. PDO apples were collected in Val di Non and Val di Sole (Trentino); PGI apples from South Tyrol were collected in Val Venosta (including two orchards in the surrounding of Merano, no. 22 and 24) (Table 1), in the surrounding of Bressanone and southern of Bolzano (later referred to as ‘Val d'Adige’), whereas PGI apples from Lombardia were collected in Valtellina. Non‐GI apples came from 13 orchards located in Emilia Romagna, Lombardia, Piemonte and Veneto. More details, including geographical coordinates and altitude of each orchard, are provided in Table 1.

**Table 1:** Main details for each sampling site

Orchard number	Label^a^	Region	District	Location	GPS coordinates		Altitude m a.s.l.	Sampling year
					Latitude	Longitude		
1	PDO Val di Non	Trentino‐South Tyrol	Val di Non	Brez	46.424323	11.105225	701.7	2017
2	PDO Val di Non	Trentino‐South Tyrol	Val di Non	Coredo	46.344349	11.093484	842.3	2017, 2018
3	PDO Val di Non	Trentino‐South Tyrol	Val di Non	Mechel	46.348412	11.021362	725.9	2017, 2018
4	PDO Val di Non	Trentino‐South Tyrol	Val di Non	Revò	46.386955	11.058860	660.4	2017
5	PDO Val di Non	Trentino‐South Tyrol	Val di Non	Rumo	46.432930	11.030728	885.7	2017, 2018
6	PDO Val di Non	Trentino‐South Tyrol	Val di Non	Termon	46.268034	11.037990	556.9	2017
7	PDO Val di Non	Trentino‐South Tyrol	Val di Non	Tuenno	46.318183	11.034340	592.2	2017
8	PDO Val di Non	Trentino‐South Tyrol	Val di Sole	Presson	46.332050	10.876865	809.6	2017, 2018
9	PGI South Tyrol	Trentino‐South Tyrol	Bressanone	Albes	46.681682	11.630275	558.2	2017, 2018
10	PGI South Tyrol	Trentino‐South Tyrol	Bressanone	Elvas	46.743737	11.668427	883.6	2017, 2018
11	PGI South Tyrol	Trentino‐South Tyrol	Bressanone	Naz	46.753018	11.683977	877.5	2017
12	PGI South Tyrol	Trentino‐South Tyrol	Bressanone	Sarnes	46.685335	11.641758	575.4	2017
13	PGI South Tyrol	Trentino‐South Tyrol	Val d'Adige	Binnenland	46.343396	11.279116	215.0	2017, 2018
14	PGI South Tyrol	Trentino‐South Tyrol	Val d'Adige	Egna	46.302854	11.258809	218.0	2017
15	PGI South Tyrol	Trentino‐South Tyrol	Val d'Adige	Laimburg	46.384960	11.290982	226.3	2017, 2018
16	PGI South Tyrol	Trentino‐South Tyrol	Val d'Adige	Laives	46.434610	11.336136	232.9	2017, 2018
17	PGI South Tyrol	Trentino‐South Tyrol	Val d'Adige	Ora	46.375390	11.302930	224.4	2017
18	PGI South Tyrol	Trentino‐South Tyrol	Val d'Adige	Salorno	46.254468	11.237509	210.3	2017
19	PGI South Tyrol	Trentino‐South Tyrol	Val d'Adige	Vadena	46.411754	11.307478	227.4	2017
20	PGI South Tyrol	Trentino‐South Tyrol	Val Venosta	Castelbello	46.622175	10.919891	594.7	2017, 2018
21	PGI South Tyrol	Trentino‐South Tyrol	Val Venosta	Corces	46.627292	10.755757	723.8	2017
22	PGI South Tyrol	Trentino‐South Tyrol	Val Venosta	Fragsburg	46.637184	11.196591	720.5	2017
23	PGI South Tyrol	Trentino‐South Tyrol	Val Venosta	Plaus	46.650295	11.043524	525.6	2017
24	PGI South Tyrol	Trentino‐South Tyrol	Val Venosta	Sinigo	46.643324	11.180739	278.7	2017, 2018
25	PGI South Tyrol	Trentino‐South Tyrol	Val Venosta	Sluderno	46.659413	10.579095	910.2	2017, 2018
26	PGI Valtellina	Lombardia	Valtellina	Berbenno in Valtellina	46.166111	9.767778	300.0	2017
27	PGI Valtellina	Lombardia	Valtellina	Sernio	46.219414	10.206319	694.0	2017
28	PGI Valtellina	Lombardia	Valtellina	Tresivio	46.178563	9.961554	600.0	2017, 2018
29	Non‐GI	Emilia Romagna	Pianura Padana	Bagnolo	44.231759	12.098541	20.19	2017, 2018
30	Non‐GI	Emilia Romagna	Pianura Padana	Borgo Manara	44.766111	12.195278	−1.3	2017
31	Non‐GI	Emilia Romagna	Pianura Padana	Roncadello	44.280714	12.047838	16.41	2017
32	Non‐GI	Emilia Romagna	Pianura Padana	Sant’Agostino	44.775323	11.347170	13.02	2017, 2018
33	Non‐GI	Emilia Romagna	Pianura Padana	San Bartolo	44.364613	12.190140	3.45	2017
34	Non‐GI	Emilia Romagna	Pianura Padana	Sesto Imolese	44.458619	11.732518	18.8	2017
35	Non‐GI	Emilia Romagna	Pianura Padana	Volania	44.733893	12.162780	−2.1	2017
36	Non‐GI	Veneto	Pianura Padana	Ceregnano	45.049166	11.88493	4.0	2017, 2018
37	Non‐GI	Veneto	Pianura Padana	Eraclea	45.615708	12.741126	−1.0	2017
38	Non‐GI	Veneto	Pianura Padana	Jesolo	45.546268	12.712247	0.8	2017, 2018
39	Non‐GI	Lombardia	Pianura Padana	Corzano	45.453467	10.002428	95.4	2017, 2018
40	Non‐GI	Piemonte	Pianura Padana	Savigliano	44.679479	7.688665	296.5	2017, 2018
41	Non‐GI	Piemonte	Pianura Padana	Spinetta	44.382016	7.583676	530.4	2017, 2018

a PDO, protected designation of origin; PGI, protected geographical indication; Non‐GI, without geographical indications.

### Sampling campaign

In 2017, apples were collected from 41 orchards and, within each orchard, three fruits per tree were harvested from ten randomly selected trees. Fruits were sampled at a height of 1–2 m from the ground. The three apples were grouped together to create a bulk sample for each tree (*n* = 410). In 2018, the sampling campaign was repeated in 20 of the previously investigated orchards, and both apple and soil samples were collected. The orchards for the 2018 sampling were selected to cover the whole sampling area and the whole range of the ^87^Sr/^86^Sr ratio of the apples collected in 2017 (Table 1). The apple sampling was repeated following the same procedure adopted in 2017 using five trees per each orchard (*n* = 100). Beneath the canopy of each sampled tree, a soil core (at a depth of 10–40 cm from the surface) was collected at a distance of 30 cm from the tree trunk using a soil auger (one‐piece Edelman type). Each soil core was treated as a bulk sample (*n* = 100).

### Sample preparation

- Sr extraction from soil samples

Soil samples were dried at 65 °C for 48 h and sieved at 2 mm. All the root pieces were manually removed. The Sr‐bioavailable fraction was extracted with NH_4_NO_3_ in accordance with the official method DIN ISO 19730.^14^

- Acid digestion of apples

On each of the three apples collected per tree, the peel was manually removed using a peeler. Then, the three central disks (approximate thickness of 1 cm) were isolated from lateral disks and the fruit core was removed. The disks were freeze‐dried and powdered together to create a bulk sample per tree. Each sample (0.5 g) was digested in a Milestone UltraWAVE apparatus, adding 5 mL of sub‐boiled HNO_3_ (14 mol L^‐1^) and 1 mL of H_2_O_2_. After digestion, samples were filtered with PTFE filters (0.45 μm).

### Sr/matrix separation

Sr/matrix separation was performed with a Sr‐specific resin (TrisKem International), in accordance with the methods proposed by Swoboda *et al*.^9^ and Durante *et al*.,^16^ with slight modification as described in a previous study.^17^ The efficiency of the Sr/matrix separation was verified at the ICP‐MS, and then the final concentration of the Sr solutions was adjusted to 200 ng g^−1^ for soil‐derived samples and 20 ng g^−1^ for apple‐derived samples.

### ICP‐MS and MC ICP‐MS analysis

Calcium, Rb and Sr were quantitatively analyzed using an inductively coupled plasma mass spectrometer (iCAP Q ICP‐MS) (Thermo Scientific, Bremen, Germany) equipped with an autosampler ASX‐520 (Cetac Technologies Inc., Omaha, NE, USA). The calibration curve was prepared in the range 0.025–250 ng g^−1^ for Rb and Sr, and in the range 0.025–25 μg g^−1^ for Ca, including instrumental blanks. In the investigated range, the calibration curves showed good linearity (*r* ^2^ > 0.9996). A standard solution of Sc, Ge and Y was used as internal standard. The accuracy of the instrument was monitored measuring the certified reference material TMDA‐64.3 as a QC at different dilutions. On average, element concentrations in the QC solutions ranged between 90% and 110% of the certified value. The operating conditions were reported in a previous study.^15^

The ^87^Sr/^86^Sr was measured with a double‐focusing MC ICP‐MS (Neptune Plus™; Thermo Scientific). Soil samples were measured in wet‐plasma conditions, whereas apple samples in dry‐plasma conditions [CETAC Aridus apparatus as aerosol drying unit (Teledyne, Omaha, NE, USA) and a Jet sample cone + Ni ‘H' skimmer cone (Thermo Scientific)]. Instrument configuration, typical operating conditions and data corrections as were reported in previous studies.^15^, ^17^

The instrument was tuned daily and its accuracy was determined analyzing the SRM 987, with a certified ^87^Sr/^86^Sr ratio at the beginning, at the end and at every block of samples in the sequence bracketed with a blank solution.^18^, ^19^ Replicated measurements of the SRM 987 (*n* = 114), both in wet and dry plasma configuration, provided an average ^87^Sr/^86^Sr ratio of 0.710257 ± 0.000014 (with the uncertainty expressed as twice the standard deviation, 2*sd*) within the measuring period of this study, in agreement with the instrumental precision reported by other authors.^11^, ^20^ This result is also consistent with both the certified and ‘generally accepted’ value, respectively equals to 0.71034 ± 0.00026 and 0.710263 ± 0.000016 (with the uncertainty expressed as 2 *s*), and provides an estimate of the instrument long‐term precision and accuracy. Considering the repeatability of the analytical procedure, several independent aliquots of apples (*n* = 15) and soil samples (*n* = 19) were prepared and analyzed, in accordance with the procedure described above. The relative standard deviations obtained were, respectively, 0.0018% and 0.0025% for apple and soil samples.

### Statistical analysis

Statistical analysis was applied to evaluate the results of the ^87^Sr/^86^Sr analysis at different levels. Kruskal–Wallis one‐way analysis of variance and a Dunn post‐hoc test (with Bonferroni correction) were applied to compare mean values from ^87^Sr/^86^Sr analysis of each cultivation district. The non‐parametric analysis of variance was applied because the groups included in the comparison did not have an equal number of samples and at least one of the conditions enabling parametrical tests (normal distribution, variance homogeneity) was not satisfied. For this comparison, the three main cultivation districts of South Tyrol (Val Venosta, Bressanone area and Val d'Adige) were treated as separated groups, together with the other districts. Linear correlation analysis and a two‐tailed *t* test for paired samples was applied to evaluate the year‐to‐year (2017 *versus* 2018) variability of the ^87^Sr/^86^Sr ratio in apple samples. Linear correlation was also applied to the results of the ^87^Sr/^86^Sr ratio analysis of the apple and soil samples. Cluster analysis, according to Euclidean distances and Ward's hierarchical method, was used as an unsupervised technique to divide the dataset into different groups with the lowest internal variance and the highest difference between groups based solely on the ^87^Sr/^86^Sr ratios. *P* < 0.05 was considered statistically significant. The statistical analysis was performed using the computing environment R (R Core Team, 2016).

## RESULTS AND DISCUSSION

### Year‐to‐year ^87^Sr/^86^Sr ratio variability and correlation with the soil ^87^Sr/^86^Sr ratio

**Figure 2:**
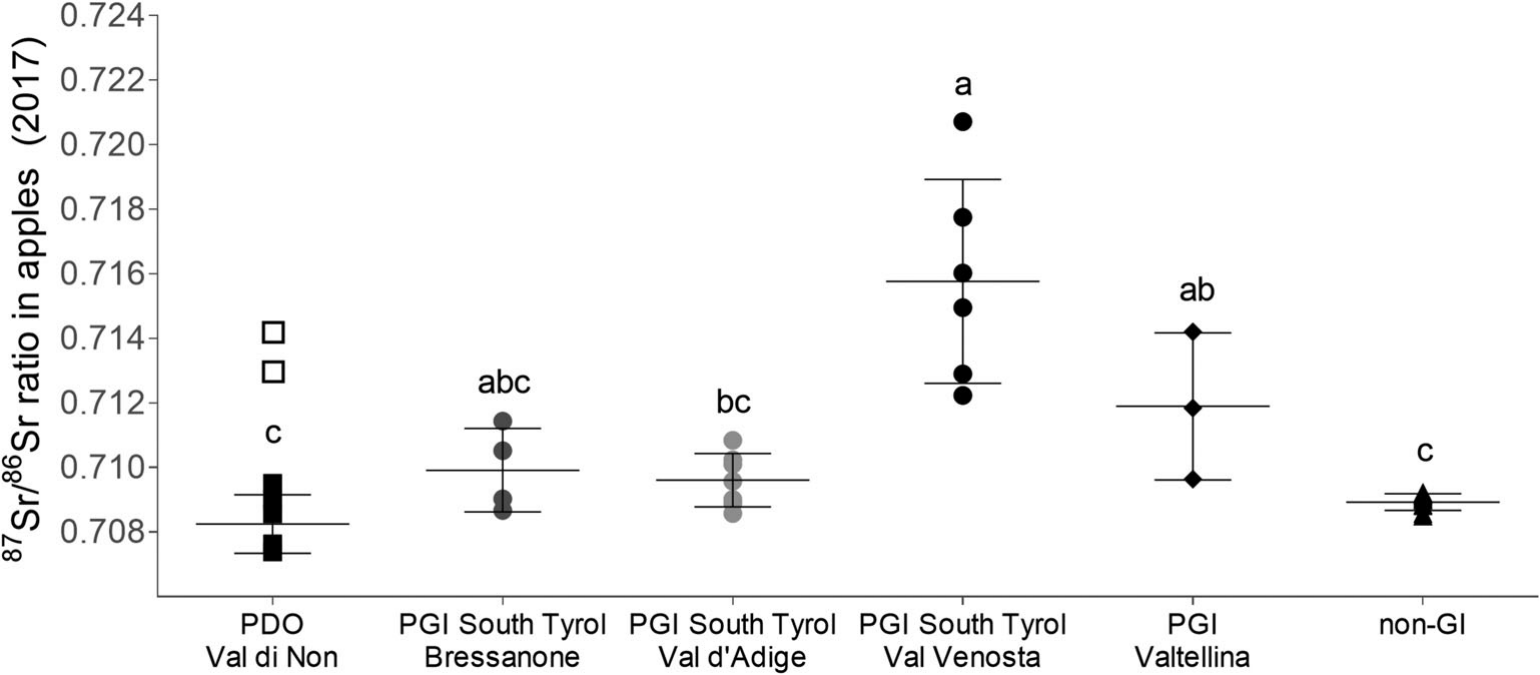
^87^Sr/^86^Sr ratios measured in apple orchards (2017) grouped according to the cultivation district. Horizontal lines represent the mean ± SD of each district. Each symbol represents one orchard and is the mean of 10 apple samples. Open squares represent the two orchards in Val di Non excluded from the mean calculation and the analysis of variance among cultivation districts. Different letters indicate a significant difference among districts (*P* < 0.05).

The ^87^Sr/^86^Sr ratio of apples collected in 2017 ranged from a minimum of 0.7074 ± 0.0002 (mean ± SD) to a maximum of 0.7207 ± 0.0016 (Fig. 2). PGI apples from Val Venosta had, on average, the highest ^87^Sr/^86^Sr ratio (0.7158 ± 0.0032), although the six orchards stretched along a large range of values (Fig. 2). The ratio of apples from Val Venosta was not statistically different from the PGI apples originating from Valtellina (0.7119 ± 0.0023) or the Bressanone area (0.7099 ± 0.0013). At the same time, apples from Val Venosta differed from PGI apples from Val d'Adige (0.7096 ± 0.0008) and from the non‐GI apples cultivated in other apple districts in Northern Italy. The latter, despite the large extension of the sampling area that included orchards from five different regions (Fig. 1), showed a quite narrow variability of the ^87^Sr/^86^Sr ratio (0.7089 ± 0.0003). Non‐GI apples had isotope ratios significantly lower than the ratio of Valtellina too. PDO apples from most orchards in Val di Non fell in a narrow range of values, between 0.7074 and 0.7095, although two orchards (no. 5 and 8), located at the border with Val Venosta, on a different rock type (Fig. 1), had higher values (0.7136 ± 0.0009) (Fig. 2). When these two orchards were excluded from the statistical analysis, it was found that apples from Val di Non had significantly lower ^87^Sr/^86^Sr ratio than those from Val Venosta and Valtellina. For a complete overview of the results, the whole dataset is reported in the Supporting information (Table S1).

**Figure 3:**
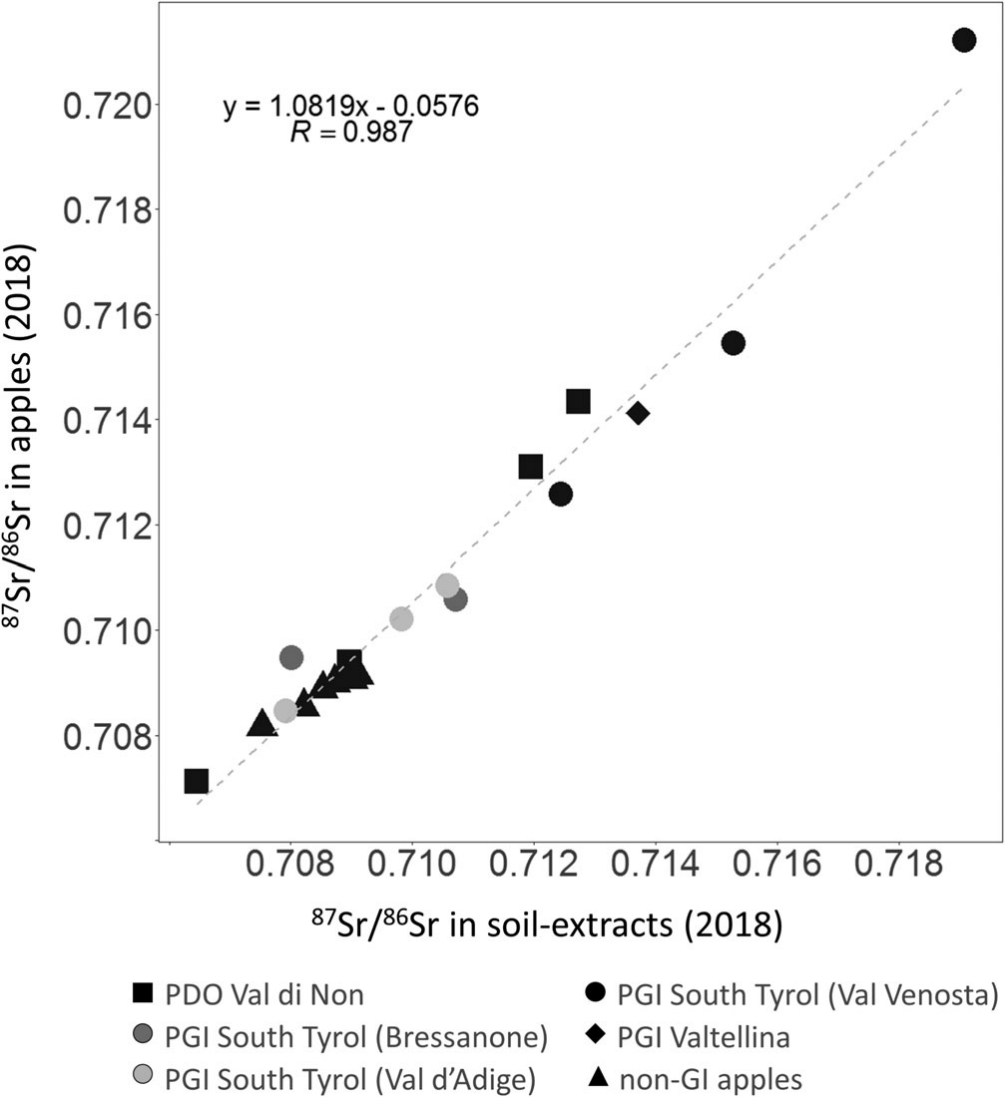
Linear correlation between the mean ^87^Sr/^86^Sr ratios of apple and soil extracts. Data are grouped according to the cultivation district.

Figure 3 shows the correlation between the ^87^Sr/^86^Sr ratio of soil extracts and that of the corresponding apples collected during the second sampling campaign (see Supporting information, Table S2). The isotopic composition of apple and soil extracts collected in 2018 was in the range 0.7074–0.7207 and 0.7065–0.7191, respectively. As shown in Fig. 3(B), a strong positive correlation between ^87^Sr/^86^Sr ratios of apple and soil samples was found (*r* = 0.99, *P* < 0.01). Such agreement has been already reported previously,^11^, ^21^, ^22^ and confirms the dependence of the apple ^87^Sr/^86^Sr ratios on that of the soil. Moreover, a good overlap was found between the range of variability of the ratios for the apple and soil samples within each orchard, as attested by the SDs reported in Supporting information (Table S2).

**Figure 4:**
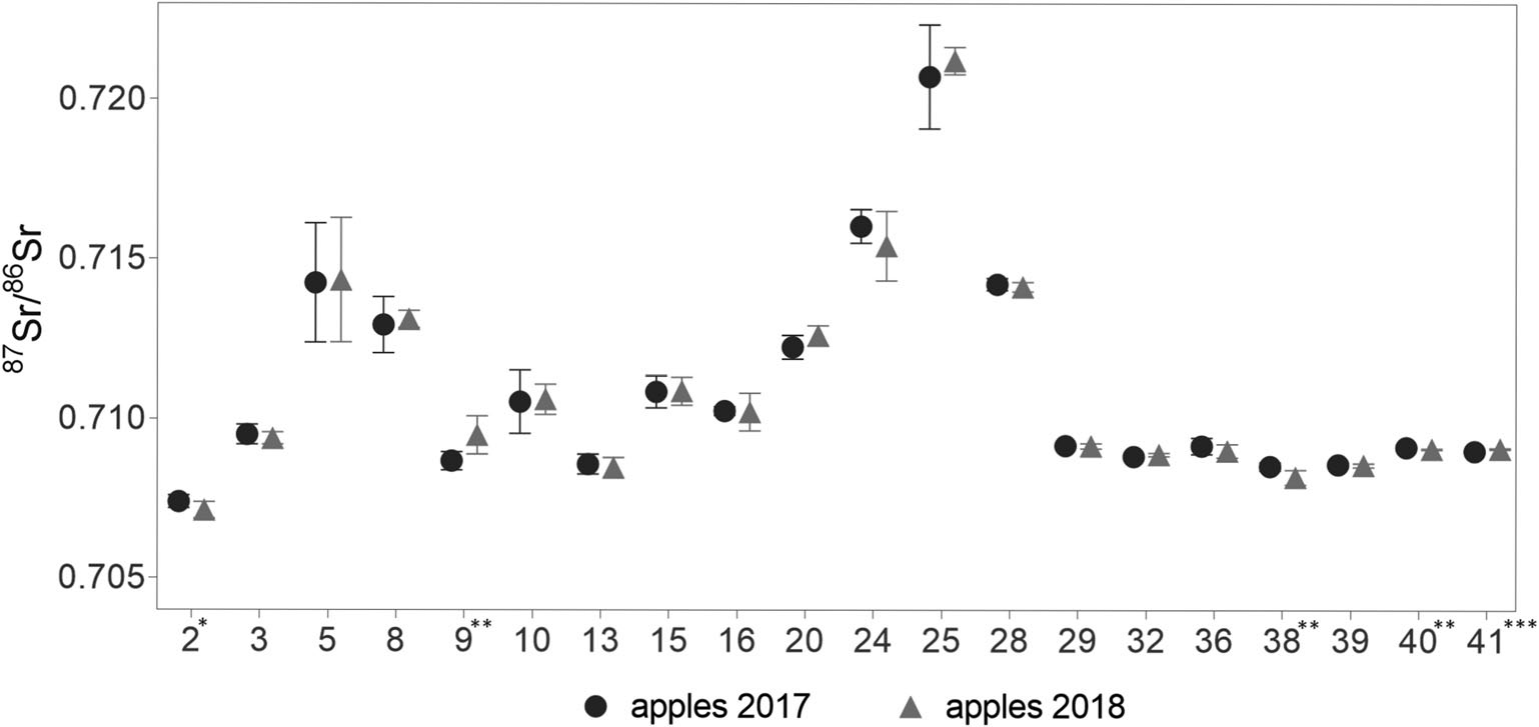
Comparison between the mean ^87^Sr/^86^Sr ratios of apples from two consecutive years (2017 and 2018) for each orchard. Orchards are numbered according to Table 1. Asterisks denote statistical significance: *P < 0.05, **P < 0.01, ***P < 0.001.

In Fig. 4, a comparison of the ^87^Sr/^86^Sr ratio of apples collected in the two consecutive years is provided. Considering only the 20 orchards sampled both in 2017 and 2018, the ^87^Sr/^86^Sr ratio was in the range 0.7074–0.7207 in 2017 and 0.7071–0.7212 in 2018. The correlation between data was very high (*r* > 0.996, *P* < 0.01) and, in all but five orchards, the data corresponded perfectly (Fig. 4). This confirms that the ^87^Sr/^86^Sr ratio is a rather stable indicator characterized by temporal invariance.^23^ Comparing the mean ratios for apples collected in 2017 and 2018, significant differences between the 2 years were found for five orchards (no. 2, 9, 38, 40 and 41) (Fig. 4). For two of them (no. 40 and 41), the difference between the two ratios was significant despite a low difference between the two mean ratios that was 0.00005 and 0.00008, respectively. Moreover, the ^87^Sr/^86^Sr ratio of apples within the two orchards was highly homogeneous, with SDs lower than 0.000025, on average, for both years. This confirms that, for orchards characterized by high intra‐orchard homogeneity, such as orchards no. 40 and 41, even extremely low differences between mean ratios can be significant.^17^

### Interpretation of the apple ^87^Sr/^86^Sr ratio based on geolithological information

**Figure 5:**
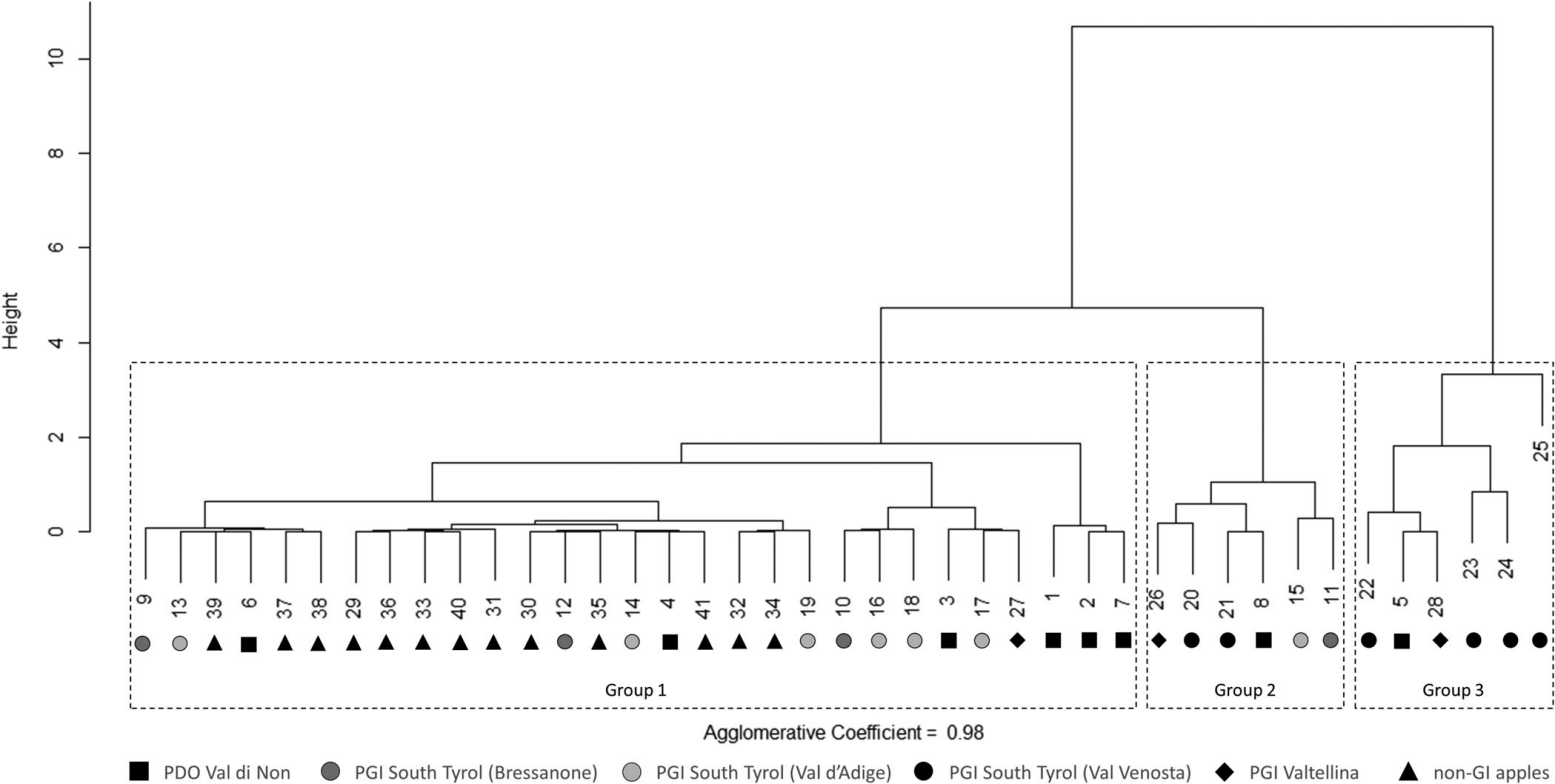
Dendrogram of cluster analysis based on the ^87^Sr/^86^Sr ratios of apple samples.

The cluster analysis was applied to group the orchards based solely on similarities of their ^87^Sr/^86^Sr ratio. In Fig. 5, the outcome of the hierarchical procedure is provided (agglomerative coefficient = 0.98). At a distance of 4, three different groups could be identified, suggesting that apples are not separated according to geographical or commercial borders. The first group (i.e. the largest) includes 29 orchards producing PGI apples from South Tyrol (*n* = 6 from Val d'Adige and n = 3 from Bressanone), PGI apples from Valtellina (*n* = 1), PDO apples from Val di Non (*n* = 6) and all the orchards producing non‐GI apples (*n* = 13). The second group consists of one orchard each from Bressanone, Val d'Adige, Val di Non, Valtellina and two orchards from Val Venosta. The third group includes one orchard from Val di Non and Valtellina and four orchards from Val Venosta.

To better explain these results, the relationship between the apples and their area of cultivation was examined. Because there was a good correlation between soil and apple ^87^Sr/^86^Sr ratio in the investigated sampling sites (Fig. 3), the results of the cluster analysis were compared with geolithological information available for each area (Fig. 1). The bioavailable Sr fraction in the soil derives largely from that of the minerals present in the bedrock, from which Sr is released via a mechanism of differential weathering.^24^, ^25^ Therefore, the soil ^87^Sr/^86^Sr represents a weighted average of the local mineral composition. The ^87^Sr/^86^Sr ratio presently measured in Sr‐bearing minerals results from the sum of the primordial and radiogenic ^87^Sr and depends on three pivotal factors: the initial ^87^Sr/^86^Sr, the Rb/Sr ratio and the age of the mineral. In general, minerals showing the lowest ratios are carbonates and plagioclase feldspars, whereas the highest ratios are measured in K‐feldspars and micas.^8^

As already noted, all the orchards producing non‐GI apples (no. 29–41) belong to the first cluster. These orchards are located mostly in a vast basin, the Pianura Padana, characterized by sedimentary deposits that developed alluvial soils. This basin is bounded by mountain chains (Alps to the North and Apennine to the South) and it expands to the Adriatic Sea. The Po river, its tributaries and a series of Apennine rivers create the local fluvial network. Lithological analysis revealed that the sediments mostly characterizing the alluvial plain are limestones, felsic intrusive rocks, dolomites, mafic and ultramafic detritus, sandstones and marls, and littoral and shallow‐marine deposits, with a cyclic sedimentation pattern being described along the plain.^26^, ^27^, ^28^ The ^87^Sr/^86^Sr ratios of apples measured in the present study (0.7089 ± 0.0003) fall within a range of values comparable to the range measured in the Modena district and adjacent provinces in other studies, which reported ratios of around 0.708–0.710,^16^, ^21^, ^23^ and are compatible with the lithological description of the Pianura Padana.

The first cluster also includes six orchards from Val di Non (no. 1–4, 6 and 7). The apple cultivated in Val di Non were mainly characterized by low isotope ratios (Fig. 2; see also Supporting information, Table S1). Low values of ^87^Sr/^86^Sr ratio can be ascribed to the position of the orchards cultivated on soil that developed over colluvial fans of carbonate sediments from the Triassic period. Indeed, this area is characterized by the presence of carbonate rocks (calcite and dolomite) that formed in a marine environment through the precipitation of calcium carbonate (shell debris, fecal material, coral fragments). In a study conducted by Faure *et al*.^29^ in Val Camonica (Lombardia) on carbonate rocks, ^87^Sr/^86^Sr ranged between 0.7070–0.7085, and it was stated that these values are representative of the Sr present in the ocean at the deposition time. Willmes *et al*.^30^ measured similar ^87^Sr/^86^Sr ratios for dolomites and limestones in France (0.707–0.710).

In the lower part of the Val d'Adige, orchards are cultivated on soils formed from quaternary deposits. The whole valley was modeled by the effect of multiple glaciations that occurred in the Pleistocene and the fine‐grained sediments (sand, silt, clay) filling the valley are mainly of alluvial and glacio‐alluvial origin, locally mixed with detritus originating the lateral slopes.^31^, ^32^, ^33^ The surrounding area is characterized by the presence of the ‘Complesso Vulcanico Atesino’, formed during the early Permian and covering an area of 2000 km^2^. The ‘Formazione di Ora’ is the most recent volcanic deposit of the area and covers an area of approximately 1500 km^2^ that includes both sides of the Val d'Adige South from Bolzano and is mainly composed of rhyolitic lapilli‐tuff.^34^ As a result, the ^87^Sr/^86^Sr in Val d'Adige tends to show values close to those typical for alluvial plains, although they showed higher variability with respect to the ratios in apple and soil samples (Figs 2 and 3; see also Supporting information, Table S1), in agreement with the geolithological information.

All of the orchards located in the Bressanone area (no. 9, 10 and 12) are grouped in the first cluster, except one that belongs to the second cluster because it is characterized by a slightly higher ^87^Sr/^86^Sr. The cultivation area around Bressanone lays on a crystalline basement called Brixen Quarzphyllite (Variscan orogeny and low‐grade metamorphism), with a quite homogeneous lithology and the prevalence of minerals with a relatively low Rb/Sr ratio,^34^ which explains why the ^87^Sr/^86^Sr ratio of apples was rather low (0.7099 ± 0.0013 in apples, 0.7094 ± 0.0019 in soil extracts).

Finally, the high ^87^Sr/^86^Sr ratios measured in apples produced in the orchards of the second and third cluster and located in Valtellina (0.7119 ± 0.0023), Val Venosta (0.7158 ± 0.0032), and two orchards of Val di Non (at the border with Val Venosta) (0.7136 ± 0.0009) can be related to the vast geological domain of the Austroalpine unit, a crystalline basement further divided into different subunits that shows a complex polymetamorphic history.^35^ Here, metamorphic rocks are mainly present, including feldspars, migmatites, pegmatites and orthogneisses (granite gneisses). Bioavailable Sr deriving from these minerals shows relatively high isotope ratios. Willmes *et al*.^30^ reported values between 0.710–0.723 and 0.715–0.721 for lithological areas rich in migmatite and orthogneiss rocks, respectively. The soil that developed on coarse‐grained sediments within this area is characterized by a relatively high heterogeneity and, hence, by a large range of ^87^Sr/^86^Sr ratio. Local differences and fluctuations in the ^87^Sr/^86^Sr ratio can be also ascribed to anthropogenic activity, as documented for certain areas of Val Venosta (e.g. the area of orchard no. 21), where fine landfill material was moved during the 1950s. The ratios of the two orchards located in the surrounding of Merano (no. 22 and 24) were also high (0.7155 ± 0.0008 in apples) (Table S1). In this case, the determining factor explaining the ^87^Sr/^86^Sr ratio in the apple orchards can be related to the presence of magmatic rocks (basaltic andesites, andesites, dacites, rhyodacites and rhyolites in different proportions) covering the metamorphic basement. In this area, the ^87^Sr/^86^Sr of these rocks can vary from 0.707 as measured in quarts norite to 0.744 as measured in granites, demonstrating the hybrid nature of these magmatic rocks.^36^, ^37^

Further investigations, including grain size and petrographic analysis, would be help for highlighting local correlations between the Sr‐bioavailable fraction and the mineral composition. The available geolithological features combined with data from the literature already provided a good explanation of the measured ratios and clarify the formation of clusters that do not reflect commercial or geographical divisions.

## CONCLUSIONS

The suitability of the ^87^Sr/^86^Sr ratio as soil‐derived traceability marker was tested to distinguish the production of Italian PDO and PGI apples (cv. Golden Delicious) from apples cultivated in other districts of Northern Italy. The ^87^Sr/^86^Sr ratios of apples collected from the main apple production districts and the ^87^Sr/^86^Sr ratios of the soil bioavailable Sr fraction were highly correlated. The results of the ^87^Sr/^86^Sr ratio analysis for a year‐to‐year comparison indiacted that this parameter is rather stable, showing a low temporal variability. The ^87^Sr/^86^Sr ratio of apples also agree with the geolithological features of the different cultivation areas. We can therefore confirm that the ^87^Sr/^86^Sr ratio is an excellent marker for studies of food traceability. However, whenever the cultivation districts had a similar soil Sr isotope composition, a complete separation of the apple districts based solely on the ^87^Sr/^86^Sr ratio of the apples was not possible. The apples produced in the Po valley had a rather homogeneous ^87^Sr/^86^Sr ratio, despite the relatively large size of the Po valley, because of the soil ^87^Sr/^86^Sr ratio homogeneity and also differed from those from Val Venosta and Valtellina. By contrast, PDO and PGI apples produced in relatively small mountain valleys show a higher variability of the ^87^Sr/^86^Sr ratio because the orchards are sometimes planted on soils from different bedrocks. In conclusion, the ^87^Sr/^86^Sr ratio has the potential to distinguish between different cultivation areas as long as these areas are characterized by geolithological differences. The present study represents first effort to enhance the tutelage of high‐quality apples cultivated in the Italian districts using objective and reliable analytical tools based on firm soil features. In the perspective of apple traceability, it would be useful to include other parameters related to geographic origin in the discrimination analysis; for example, multi‐element analysis or the light element isotope ratios.

## CONFLICT OF INTERESTS

The authors declare that they have no conflicts of interest.

## Supporting information


**Table S1** **.** Results of the 87Sr/86Sr isotope ratio for apples collected in 2017.
**Table S2.** Results of the 87Sr/86Sr isotope ratio for apples and soil extracts collected in 2018.
