# Supplementary material for: Sr isotope composition of Golden Delicious apples in Northern Italy reflects the soil 87Sr/86Sr ratio of the cultivation area
Source: J Sci Food Agric. 2020 Apr 25;100(9):3666–74. doi: 10.1002/jsfa.10399 (PMC7384160; doi:10.1002/jsfa.10399)
Supplement: Supplementary file 1 — Table S1 . Results of the 87Sr/86Sr isotope ratio for apples collected in 2017. Table S2. Results of the 87Sr/86Sr isotope ratio for apples and soil extracts collected in 2018. [file JSFA-100-3666-s001.pdf]

Supporting Information

Sr isotope composition of Golden Delicious apples in Northern Italy reflects soil $^{87}Sr/^{86}Sr$ ratio of the cultivation area

Authors

Agnese Aguzzoni[1]*, Michele Bassi[2], Emanuela Pignotti[2], Peter Robatscher[2], Francesca Scandellari[1], Werner Tirler[3], Massimo Tagliavini[1]

Affiliations

[1] Free University of Bozen-Bolzano, Piazza Università 1, 39100 Bozen-Bolzano, Italy

[2] Laimburg Research Centre, Laimburg 6, Pfatten-Vadena, 39040 Auer-Ora, Italy

[3] Eco-Research srl, Via Luigi Negrelli, 13, 39100 Bozen-Bolzano, Italy

*Corresponding author – E-mail: agnese.aguzzoni@unibz.it

Table S1. Results of the $^{87}Sr/^{86}Sr$ isotope ratio for apples collected in 2017.

site	sampling site	label	$^{87}Sr/^{86}Sr$	sd	n
1	Brez	PDO - Val di Non	0.70763	0.00023	10
2	Coredo	PDO - Val di Non	0.70739	0.00020	10
3	Mechel	PDO - Val di Non	0.70950	0.00031	10
4	Revò	PDO - Val di Non	0.70900	0.00026	10
5	Rumo	PDO - Val di Non	0.71424	0.00187	10
6	Termon	PDO - Val di Non	0.70857	0.00017	10
7	Tuenno	PDO - Val di Non	0.70739	0.00031	10
8	Presson	PDO - Val di Non	0.71292	0.00088	10
9	Albes	PGI – South Tyrol (Bressanone)	0.70867	0.00029	10
10	Elvas	PGI - South Tyrol (Bressanone)	0.71052	0.00099	10
11	Naz	PGI - South Tyrol (Bressanone)	0.71144	0.00042	10
12	Sarnes	PGI - South Tyrol (Bressanone)	0.70903	0.00053	10
13	Binnenland	PGI - South Tyrol (Val d'Adige)	0.70857	0.00031	10
14	Egna	PGI - South Tyrol (Val d'Adige)	0.70901	0.00025	10
15	Laimburg	PGI - South Tyrol (Val d'Adige)	0.71084	0.00050	10
16	Laives	PGI - South Tyrol (Val d'Adige)	0.71024	0.00014	10
17	Ora	PGI - South Tyrol (Val d'Adige)	0.70958	0.00040	10
18	Salorno	PGI - South Tyrol (Val d'Adige)	0.71010	0.00039	10
19	Vadena	PGI - South Tyrol (Val d'Adige)	0.70889	0.00028	10
20	Castelbello	PGI - South Tyrol (Val Venosta)	0.71223	0.00037	10
21	Corces	PGI - South Tyrol (Val Venosta)	0.71289	0.00040	10
22	Fragsburg	PGI - South Tyrol (Val Venosta)	0.71495	0.00042	10
23	Plaus	PGI - South Tyrol (Val Venosta)	0.71774	0.00095	10
24	Sinigo	PGI - South Tyrol (Val Venosta)	0.71602	0.00053	10
25	Sluderno	PGI - South Tyrol (Val Venosta)	0.72071	0.00163	10
26	Berbenno in Valtellina	PGI - Valtellina	0.71184	0.00023	10
27	Sernio	PGI - Valtellina	0.70964	0.00033	10
28	Tresivio	PGI - Valtellina	0.71420	0.00019	10
29	Bagnolo	non-GI	0.70916	0.00004	10
30	Borgo Manara	non-GI	0.70906	0.00002	10
31	Roncadello	non-GI	0.70922	0.00005	10
32	S. Agostino	non-GI	0.70882	0.00006	10
33	S. Bartolo	non-GI	0.70912	0.00001	10
34	Sesto Imolese	non-GI	0.70885	0.00009	10
35	Volania	non-GI	0.70902	0.00003	10
36	Ceregnano	non-GI	0.70915	0.00026	10
37	Eraclea	non-GI	0.70850	0.00011	10
38	Jesolo	non-GI	0.70851	0.00013	10
39	Corzano	non-GI	0.70857	0.00007	10
40	Savigliano	non-GI	0.70911	0.00003	10
41	Spinetta	non-GI	0.70899	0.00003	10

Table S2. Results of the $^{87}Sr/^{86}Sr$ isotope ratio for apples and soil extracts collected in 2018.

site	label	apples			soil extracts		
		$^{87}Sr/^{86}Sr$	sd	n	$^{87}Sr/^{86}Sr$	sd	n
2	PDO - Val di Non	0.70712	0.00026	5	0.70646	0.00074	5
3	PDO - Val di Non	0.70938	0.00019	5	0.70897	0.00043	5
5	PDO - Val di Non	0.71433	0.00195	5	0.71273	0.00170	5
8	PDO - Val di Non	0.71310	0.00027	5	0.71195	0.00227	5
9	PGI - South Tyrol (Bressanone)	0.70948	0.00060	5	0.70801	0.00040	5
10	PGI - South Tyrol (Bressanone)	0.71060	0.00047	5	0.71071	0.00081	5
13	PGI - South Tyrol (Val d'Adige)	0.70846	0.00032	5	0.70791	0.00053	5
15	PGI - South Tyrol (Val d'Adige)	0.71086	0.00044	5	0.71058	0.00058	5
16	PGI - South Tyrol (Val d'Adige)	0.71021	0.00059	5	0.70982	0.00077	5
20	PGI - South Tyrol (Val Venosta)	0.71259	0.00031	5	0.71244	0.00052	5
24	PGI - South Tyrol (Val Venosta)	0.71546	0.00110	5	0.71528	0.00074	5
25	PGI - South Tyrol (Val Venosta)	0.72121	0.00043	5	0.71907	0.00045	5
28	PGI - Valtellina	0.71412	0.00015	5	0.71371	0.00033	5
29	non-GI	0.70915	0.00008	5	0.70911	0.00008	5
32	non-GI	0.70888	0.00005	5	0.70852	0.00017	5
36	non-GI	0.70900	0.00021	5	0.70873	0.00015	5
38	non-GI	0.70817	0.00023	5	0.70753	0.00019	5
39	non-GI	0.70855	0.00006	5	0.70822	0.00008	5
40	non-GI	0.70906	0.00002	5	0.70901	0.00004	5
41	non-GI	0.70907	0.00002	5	0.70892	0.00005	5
